# A Lightweight YOLO-PEGA-Based Method for Quantifying Fish Feeding Intensity

**DOI:** 10.3390/ani16030432

**Published:** 2026-01-29

**Authors:** Xinyu Ai, Shengmao Zhang, Shenglong Yang, Ai Guo, Zuli Wu, Xiumei Fan, Yumei Wu, Yongchuang Shi

**Affiliations:** 1 Key Laboratory of Fisheries Remote Sensing, Ministry of Agriculture and Rural Affairs, East China Sea Fisheries Research Institute, Chinese Academy of Fishery Sciences, Shanghai 200090, China; 15937343001@163.com (X.A.);; 2 School of Information Engineering, Dalian Ocean University, Dalian 116023, China

**Keywords:** feeding intensity, YOLO11, *large yellow croaker*, attention mechanism

## Abstract

In aquaculture production, manual or fixed-schedule feeding often fails to match the real-time feeding level of fish schools, and overfeeding can lead to feed wastage and water-quality deterioration, which has become a major bottleneck for both large-scale farming efficiency and environmental sustainability. During feeding, intense competition and jumping behaviors generate splashes of varying magnitudes, which can serve as an indirect visual proxy for hunger intensity. In this study, we constructed a frame-level splash-annotated dataset and performed data preprocessing. Building upon YOLO11 pretrained weights, we introduced a P2–P5 four-scale detection head to enhance small-splash recognition, injected EGMA into the backbone C3k2 blocks, and replaced stride-2 downsampling convolutions with a three-branch ADown operator. On the validation set, the proposed YOLO11-PEGA achieved a precision of 0.86 and a recall of 0.80, with mAP@0.5 exceeding 0.80 and mAP@0.5–0.95 exceeding 0.30. Compared with the baseline model, the parameter count was reduced by 72.3%. The results demonstrate that the proposed model maintains stable detection and evaluation performance under complex environmental conditions, providing actionable decision support for feeding-threshold setting, feeding-time determination, and feed-amount adjustment.

## Simple Summary

Feeding fish based on experience or a fixed schedule often fails to reflect how hungry the fish are at a given moment. Overfeeding not only wastes feed but can also worsen water quality. During feeding, fish compete for pellets and frequently disturb the surface, producing splashes of different strengths. These visible splashes provide a practical clue to feeding intensity. In this study, we built a labeled video–image dataset of splash events and trained an automated model to detect small and weak splashes more reliably. The results show that the method achieves high detection accuracy on validation data while using a smaller model that is easier to deploy on farms. By converting splash observations into quantitative information, the approach offers actionable support for deciding when to stop feeding, how long to feed, and how much feed to deliver. This can help reduce feed waste and lower the risk of water-quality deterioration, contributing to more efficient and sustainable aquaculture.

## 1. Introduction

Fish feeding behavior provides important biological information for assessing hunger at both individual and school levels [1]. Feed allocation and feeding-activity analysis based on feeding behavior constitute core components of aquaculture management, directly influencing both production costs and the ecological environment of culture waters [2,3] and are also closely associated with fish welfare and production yield [4,5,6]. Accurate identification and quantification of fish feeding behavior, together with its integration into automated feeding decision-making, can effectively mitigate the adverse consequences of underfeeding or overfeeding, thereby achieving a dual optimization of economic returns and environmental benefits. With the rapid advances in computer vision and deep learning, integrating vision-based perception into intelligent feeding systems has become a viable pathway to enhance automation and intelligence in aquaculture [7,8]. In related studies, Yu et al. [9] proposed a simulated key point algorithm that enables key point selection and recognition of specific behaviors without requiring foreground segmentation. Hu et al. [10] improved the YOLOv4 (You Only Look Once, v4) model [11] to achieve efficient detection of underwater feed pellets, providing a useful reference for assessing the feeding status of fish schools. Zhang et al. [12] introduced a series of image preprocessing steps prior to model training and developed a squeeze-and-excitation model, as well as a feature-weighted image-representation approach to improve the recognition accuracy of feeding status. Qian et al. [13] employed a YOLO-based model and a linear regression method [14] to perform instance segmentation [15] and quantify the splash area generated during fish feeding. Wu et al. [16] leveraged deep learning to assess fish feeding intensity based on feeding-induced splash events. Du et al. [17] utilized three modalities—audio, video, and acoustics—to recognize fish feeding intensity. Feng et al. [18] proposed a lightweight 3D ResNet-Glore network to quantify fish feeding intensity. Despite these methodological advances, many studies rely primarily on data collected in laboratory or controlled settings, where scene diversity is limited and background interference is relatively weak; consequently, their generalization and engineering applicability remain constrained in complex real-world aquaculture scenarios such as marine net cages [19].

In net-cage aquaculture along the southeast coast of China, *large yellow croaker* is a key commercially important species, and its culture waters are frequently influenced by multiple factors, including natural illumination variability, refraction and reflection caused by spatially nonuniform light intensity, and multi-scale surface ripples induced by wind-field disturbances [20,21]. As a result, water-surface imaging conditions become highly complex, and conventional manual inspection or reliance on a single physical sensor is often insufficient to achieve continuous, objective, and quantitative monitoring of feeding behavior. Accordingly, this study focuses on “splash disturbances,” a water-surface visual manifestation that is tightly coupled with competitive feeding and readily observable in practice. When fish schools compete for feed, conspicuous splashes are generated, whose occurrence frequency, spatial distribution, and duration are strongly associated with group feeding activity [22]. This makes splash disturbance a practical and feasible observation variable for vision-based precision feeding [23].

**Figure 1:**
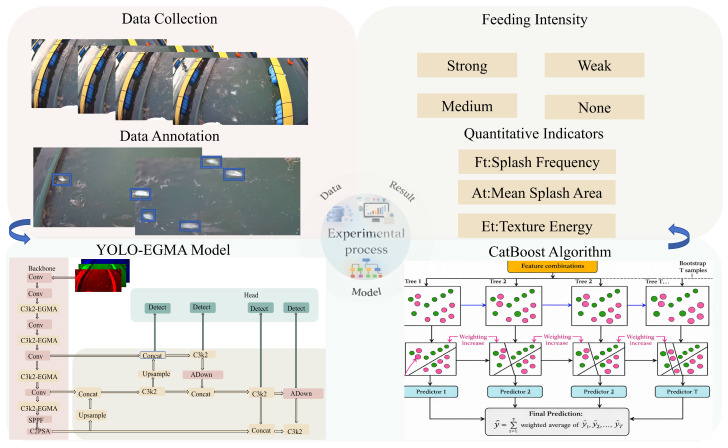
Schematic of the proposed detection workflow.

As illustrated in Figure 1, we recorded continuous videos during on-site net-cage feeding that cover multiple feeding states. Key frames were annotated on a frame-by-frame basis to label splash instances, thereby constructing a standardized dataset for model training and evaluation. The data were then split into training, validation, and test sets using video clips as the basic unit. For model development, the detector was initialized with pretrained weights for transfer learning, and attention/feature-enhancement mechanisms (e.g., EGMA) were incorporated into the YOLO backbone and feature-fusion pathway. Consequently, even under typical disturbances such as sea-surface glare, ripple noise, and rapid variations in splash scale, the network can more stably attend to discriminative cues including splash boundaries, texture discontinuities, and localized high-frequency perturbations.

Importantly, the detection output was not limited to a discrete “splash/no-splash” decision; instead, it was further projected into an interpretable behavioral-quantification space. Within a unified temporal window, splash-event frequency characterizes whether feeding competition is dense, the mean covered area reflects the magnitude of individual splashes, and texture-disturbance energy compensates for cases where weak splashes are easily confounded with background ripples. These indicators were organized into time-series feature vectors, and CatBoost was used for nonlinear fusion and probabilistic inference. The model outputs both (i) the probability distribution over four feeding-intensity levels and (ii) a continuous intensity curve obtained via probability-expectation mapping, enabling temporal fluctuations in feeding to be represented in a manner that better aligns with the inherently continuous nature of fish feeding behavior.

Accordingly, the proposed framework enables a closed-loop transformation from raw feeding videos to online intensity estimation, providing a unified and transferable quantitative basis for real-time strategy adjustment, feedback control, and anomaly warning in automated feeding.

## 2. Materials and Methods

### 2.1. Data Acquisition and Processing

#### 2.1.1. *Large Yellow Croaker* Aquaculture Data Collection

**Figure 2:**
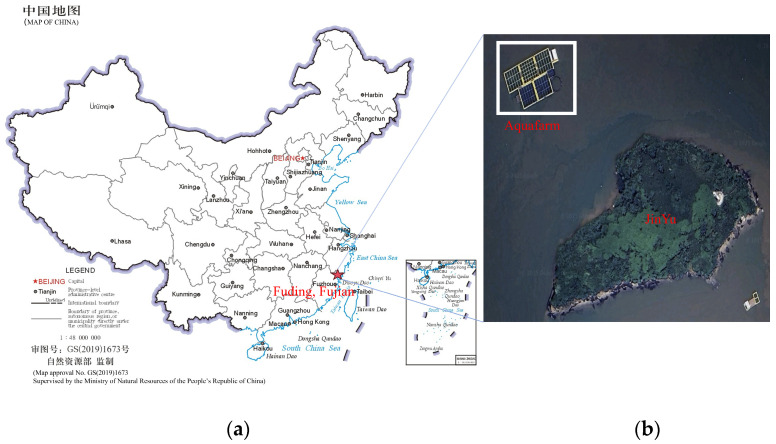
Location of the net-cage aquaculture research base. (**a**) Location of the sampling site in Fuzhou, Fujian Province, China. The map was drawn based on the official standard map provided by the Ministry of Natural Resources of the People’s Republic of China (Map approval No. GS(2019)1673). (**b**) The net-cage farming area (aquafarm) in the waters surrounding Jinyu Island. The white boxed region indicates the net-cage farming facilities.

Data were collected from the net-cage farming area at the Fujian Fuding Research Base of the East China Sea Fisheries Research Institute, located approximately 100 m northwest of Jinyu Island in Shacheng Harbor, Fuding City, Fujian Province (Figure 2). Shacheng Harbor is a natural deep-water harbor with an open water area, favorable sheltering conditions, smooth tidal exchange, and sufficient water renewal, providing a relatively stable environment for nearshore net-cage farming. The site also benefits from the natural barrier effect of surrounding islands, which mitigates direct wind–wave impacts, while maintaining good water quality that meets the ecological requirements of *large yellow croaker* for clean, oxygen-rich waters. The local waters are nutrient-rich, which is conducive to growth and development. Feeding was performed twice daily, in the early morning (approximately 05:00) and in the evening (approximately 18:00). During the feeding process, we conducted continuous on-site observations and synchronously collected water-surface splash data, providing raw data support for subsequent splash-event detection, feature quantification, and feeding-behavior extraction.

#### 2.1.2. Vision System

**Figure 3:**
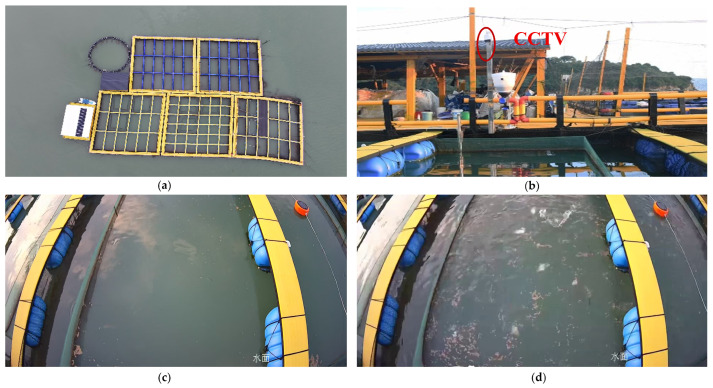
Schematic of the video acquisition system. (**a**) Top view of the net-cage farming area at the Fujian Fuding Research Base, showing the cage layout and the water-area coverage. (**b**) On-site installation location and device deployment of the video acquisition system during feeding, enabling continuous recording of water-surface disturbances within the cages. (**c**) Before feeding, the water surface is generally calm with only slight fluctuations. (**d**) After feed delivery, competitive feeding markedly intensifies surface disturbances, producing pronounced splashes and expanding ripples, which serve as the raw observational basis for subsequent splash-event detection and feeding-intensity quantification.

A video acquisition system was deployed in the net-cage farming scenario for evaluating the feeding intensity of *large yellow croaker* (Figure 3). With the net-cage area as the observation target, an industrial-grade camera was mounted approximately 1.5 m above the water surface, and the pitch angle was adjusted to ensure that splash disturbances within the covered area were fully captured in the field of view. The front-end video stream was connected to a digital video recorder for unified encoding and local storage. The recording device served solely for data logging and management and did not perform on-device inference or online analysis. Approximately 16 TB of storage was provisioned to reliably retain long-duration, multi-batch feeding data. The collected videos covered both relatively calm water-surface conditions before feeding and pronounced splashes and surface disturbances induced by competitive feeding after feed delivery, providing raw data for subsequent splash-event detection, time-series feature construction, and feeding-intensity model training.

#### 2.1.3. Dataset Construction and Annotation Protocol

Recorded videos, with a duration of approximately 20 min and a frame rate of 30 fps, were split into 1280 × 1040 RGB images in JPG format. The raw dataset D was constructed by sampling one frame per second. We then used the LabelImg annotation tool to manually record the coordinates of the minimum bounding rectangles enclosing splash regions generated during fish feeding, yielding the labeled dataset $D_{1}$ consisting of paired images and annotations. The dataset split and corresponding statistics are summarized in (Table 1). During feeding, intense aggregation, jumping, or chasing behaviors can generate splashes of varying sizes, and splash magnitude is indicative of feeding intensity [4,22]. Assessing fish feeding intensity provides an effective indicator of hunger status, which is critical for improving feed utilization and reducing water pollution.

### 2.2. Baseline: YOLO11 Overview

The YOLO family comprises a class of deep learning-based single-stage object detection frameworks that have been systematically designed and continuously evolved for real-time detection applications [24]. By adopting an end-to-end detection paradigm together with efficient feature representation and fusion strategies, these models maintain high detection accuracy while preserving fast inference and have, therefore, been widely deployed in complex vision scenarios with stringent latency requirements. In this study, we selected YOLO11 as the baseline detector [25]. Ultralytics officially released YOLO11 on 30 September 2024 [26]. This release marked a further evolution of the YOLO series in balancing speed, accuracy, and computational efficiency.

In YOLO11, the backbone replaces the conventional C2f unit with the C3k2 module to strengthen gradient flow and feature representation and further integrates the C2PSA module to enhance multi-scale semantic modeling. In addition, the redesigned spatial pyramid pooling structure works synergistically with C2PSA to improve feature diversity and context aggregation. In the neck, YOLO11 adopts a PAN-FPN architecture to bidirectionally fuse shallow details with deep semantics, thereby improving localization accuracy and scale robustness. The detection head employs a DWConv-based decoupled design to reduce parameters and computational cost, facilitating deployment on edge devices and compute-constrained platforms. Given these advantages, we selected YOLO11s as the baseline network for splash detection. Compared with the more lightweight YOLO11n variant, YOLO11s provides greater channel width and feature capacity, enabling stronger representation of small-scale splash targets with fragmented boundaries and transient textures, while still retaining favorable real-time inference characteristics suitable for on-site online monitoring in aquaculture environments.

### 2.3. Architecture of the YOLO-PEGA Splash Detection Model

**Figure 4:**
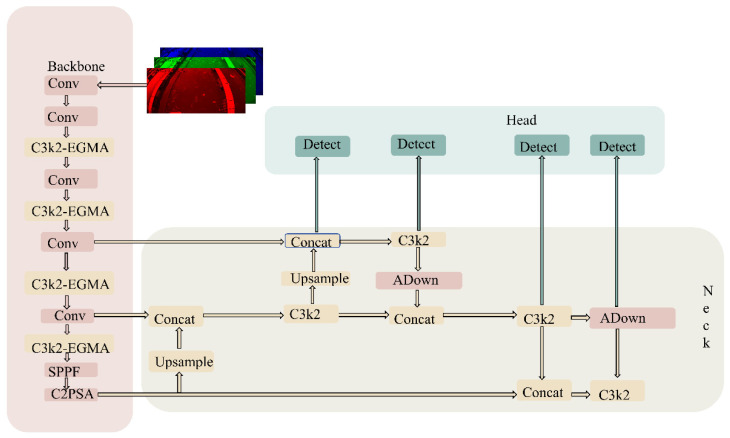
Overall architecture of the proposed YOLO-PEGA splash-detection model. The arrows indicate the direction of feature flow, and the different colors distinguish the network modules.

Although YOLO11 demonstrates strong performance in generic object detection tasks [27], further task-specific improvements are required to enhance both accuracy and efficiency when directly applying it to splash detection for fish feeding in aquaculture. This is particularly critical for small-scale splash targets, which typically exhibit unstable contrast, fragmented morphology, and blurred boundaries, and are prone to texture confusion with surface ripples, glare, and floating objects. Moreover, during repeated downsampling and cross-scale feature fusion, the fine-grained edges and transient disturbance cues of splashes can be attenuated or even lost, resulting in the coexistence of missed detections and false alarms [28]. To address these challenges, we propose YOLO-PEGA (Figure 4), a lightweight enhancement framework tailored for small-splash detection, built upon YOLO11s as the baseline with structural-level optimizations [29]. The overall architecture comprises an expanded multi-scale detection head, edge-guided backbone feature enhancement, and an improved downsampling operator, aiming to achieve more robust splash localization and recognition under complex, dynamically changing water-surface backgrounds. The backbone incorporates several targeted modifications to improve feature extraction and localization for splash targets. First, EGMA (Edge-Guided Multi-dimensional Attention) is injected into the standard C3k2 blocks at layers 2, 4, 6, and 8, forming an edge-guided enhancement unit (C3k2 + EGMA). By learning edge-saliency responses and applying edge-weighted channel statistics, this design makes feature recalibration more focused on the structural boundaries and key texture regions of splashes, thereby effectively suppressing background interference from surface ripples and glare and improving the separability and recall of weak and fragmented splashes.

Second, to reduce the loss of fine-grained spatial information during downsampling, we replace the stride-2 downsampling convolution operators with the proposed three-branch ADown module throughout the network (i.e., a unified replacement of stride-2 Conv layers in the neck/detection head). ADown fuses average pooling, max pooling, and a learnable stride-convolution branch, preserving global contextual stability while retaining salient details and edge information. This mitigates information degradation for small splash targets during scale transformations and further enhances localization stability and robustness.

Furthermore, to address the tendency of extremely small splashes to be overwhelmed in deeper feature representations, YOLO-PEGA introduces an explicit P2/4 branch in the detection head, forming a four-scale prediction structure with P2–P5 outputs. This design allows high-resolution shallow details to directly participate in prediction, strengthening early-stage representation of small-scale splashes and providing richer texture and edge cues for both classification and regression, thereby further improving overall splash-detection performance in complex aquaculture scenarios [30].

#### 2.3.1. Edge-Guided Multi-Dimensional Attention Module

In splash detection for aquaculture environments, targets are typically small, fragmented, boundary-blurred, and characterized by pronounced transient texture variations, while being heavily confounded by complex backgrounds such as surface ripples, glare, and disturbances from floating objects. Such scenarios impose stringent requirements on feature representation: fine-grained edges and localized high-frequency textures must be preserved in shallow layers, whereas “splash-like” background activations should be suppressed in deeper semantic features. However, the fixed convolutional sampling and single-channel statistics of conventional CNNs often struggle to simultaneously achieve boundary sensitivity and background robustness—particularly for weak, fragmented, or distant small splashes—thereby leading to missed detections and localization drift [31].

**Figure 5:**
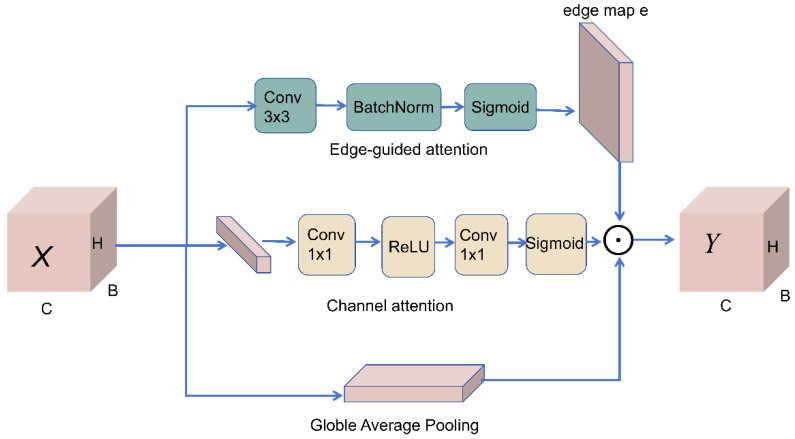
Internal architecture of the EGMA module. The arrows indicate the direction of feature/data flow and fusion within the module.

To address these issues, YOLO-PEGA introduces EGMA at key feature extraction stages of the backbone and embeds it within the C3k2 structure of YOLO11s, yielding an enhanced C3k2 + EGMA unit (Figure 5). Without a significant increase in computational overhead, EGMA leverages the synergy between edge-saliency modeling and edge-weighted channel attention to emphasize the structural boundaries and salient disturbance textures of splashes while suppressing non-target responses from the water-surface background, thereby improving separability and localization stability for small splash targets.

Mechanistically, EGMA first predicts an edge-saliency map from the input features, which explicitly models local splash discontinuities as learnable spatial priors. Guided by this edge prior, the features are reweighted to derive edge-aware global descriptors, which are then fed into a lightweight multilayer perceptron (MLP) to produce channel-attention weights for adaptive channel-wise recalibration. Finally, the channel attention and an explicit edge-enhancement term are jointly imposed on the original features, allowing the network to preserve high-level semantics while retaining fine-grained boundary cues and salient texture variations. This design improves the discriminability of splash representations under complex and rapidly varying water-surface backgrounds, thereby boosting recall for weak or fragmented splashes and enhancing boundary localization accuracy as well as temporal consistency.

The main computations of EGMA are summarized as follows (notation is consistent with the implementation; ⊙ denotes element-wise multiplication): Edge-saliency map generation: Given an input feature map $X\in R^{B\times C\times H\times W}$ a 3×3 convolution is applied followed by normalization, and the output is passed through a Sigmoid function to obtain:

$$E=\sigma (BN({Conv}_{3\times 3}(X)))\in R^{B\times 1\times H\times W}.$$

Edge-weighted feature construction:

$$X_{e}=X\;⊙\;E.$$

Edge-aware global statistics and channel attention: Global average pooling is applied to $X_{e}$, and a two-layer 1×1 convolutional MLP is used to generate the channel-wise weights:

$$G=GAP(X_{e}),A_{c}=\sigma (MLP(G))\in R^{B\times C\times 1\times 1}.$$

Jointly enhanced output: The channel recalibration and the edge-enhancement term are jointly applied to the original feature map, yielding:

$$Y=X\;⊙\;A_{c}\;⊙\;(1+E),$$

Thereby achieving multi-dimensional attention reinforcement via edge-guided channel enhancement together with spatial edge compensation.

#### 2.3.2. Lightweight Adaptive Downsampling Module

Although YOLO11s offers favorable real-time performance, cross-scale alignment in the PAN-FPN neck typically relies on stride-2 convolutions for resolution reduction. While this increases the receptive field, it can introduce irreversible smoothing of fine-grained edges and localized disturbance textures, leading to missed detections of small splashes, jitter in boundary regression, and instability in cross-scale feature fusion [32]. To this end, YOLO-PEGA proposes and incorporates ADown (Adaptive Downsampling), which replaces single-path downsampling with a multi-source complementary information-preserving strategy, thereby alleviating feature degradation during scale transformations at the structural level. Let the input feature map be denoted as: $X\in R^{B\times C_{1}\times H\times W}$. The output of ADown is given by $Y\in R^{B\times C_{2}\times \frac{H}{2}\times \frac{W}{2}}$.

To introduce complementary information without significantly increasing computational overhead, ADown allocates the output channels $C_{2}$ across three branches as follows: $C_{2}=C_{a}+C_{b}+C_{c},C_{a}=\lfloor \frac{C_{2}}{3}\rfloor ,\;\;C_{b}=\lfloor \frac{C_{2}}{3}\rfloor ,\;\;C_{c}=C_{2}-C_{a}-C_{b}$ ADown consists of three parallel paths: an average-pooling branch (global stability), a stride-convolution branch (learnable structure), and a max-pooling branch (salient detail preservation). Let ϕ(⋅) denote the SiLU activation function;

$$\begin{array}{cc} Y_{1} & =\phi \left(BN\left({Conv}_{1\times 1}^{\left(a\right)}\left({AvgPool}_{2\times 2,s=2}\left(X\right)\right)\right)\right),\\ Y_{2} & =\phi \left(BN\left({Conv}_{3\times 3,s=2}^{\left(b\right)}\left(X\right)\right)\right),\\ Y_{3} & =\phi \left(BN\left({Conv}_{1\times 1}^{\left(c\right)}\left({MaxPool}_{2\times 2,s=2}\left(X\right)\right)\right)\right),\\ Y & =Concat\left(Y_{1},Y_{2},Y_{3}\right). \end{array}$$

**Figure 6:**
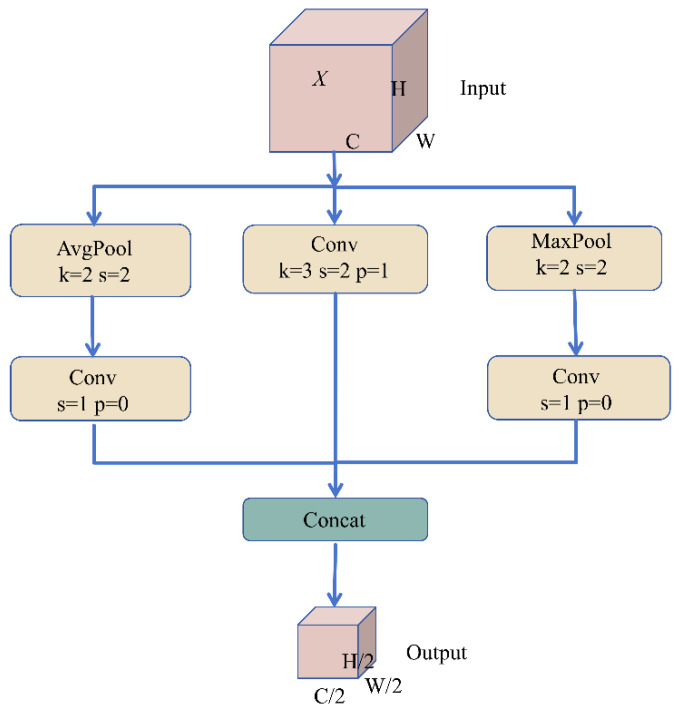
Internal architecture of the ADown module. The arrows indicate the direction of feature/data flow through the three branches and their concatenation to form the output.

This design uses average pooling and ***max pooling*** MaxPool to preserve stable background statistics and salient peak responses, respectively, while a 3×3 stride convolution on a subset of channels performs learnable structural extraction. In this way, complementary representations are retained during downsampling. With the three-way channel split, ADown can substantially reduce the parameter count and core convolutional computation compared with a full-channel 3×3 stride convolution. The internal structure of ADown is illustrated in Figure 6. Owing to these properties, ADown is better suited for deployment on resource-constrained platforms and provides computational headroom to accommodate the additional cost introduced by the P2 fine-grained detection branch.

The advantages of ADown extend beyond reduced complexity; more importantly, it improves information fidelity. The AvgPool branch provides smoother global statistics, helping suppress feature jitters induced by surface ripples and illumination reflections and thereby stabilizing cross-scale fusion. The MaxPool branch strengthens salient local responses and retains bright spots, edge discontinuities, and locally strong-gradient regions produced by splashes better—features that are particularly critical for weak and fragmented splash targets. Meanwhile, the stride-convolution branch preserves learnable structural extraction, enabling the network to adaptively encode discriminative textures under complex disturbance backgrounds. Fusing the three branches along the channel dimension yields downsampled features that jointly exhibit background robustness, detail sensitivity, and structural expressiveness, thereby mitigating information attenuation for small targets during scale transformations, improving detection recall and localization consistency, and providing higher-quality feature inputs for subsequent P2–P5 multi-scale prediction.

#### 2.3.3. P2 Detection Head Design

**Figure 7:**
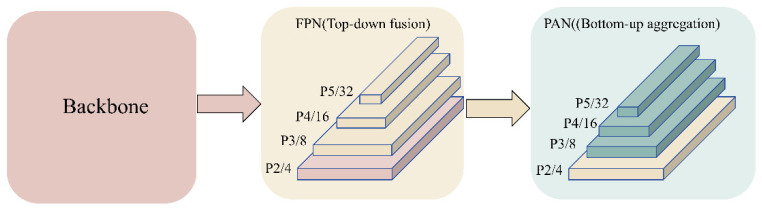
Network structure with the added P2 detection head. The arrows indicate the direction of feature transfer from the backbone to the FPN (top-down fusion) and then to the PAN (bottom-up aggregation).

The default YOLO11 detection head primarily relies on deeper feature levels (P3–P5) for prediction. For extremely small targets such as splashes, high-resolution detail cues (e.g., splash boundaries, localized bright spots, and subtle texture variations) are often smoothed or diluted in deep semantic features after repeated downsampling, leading to missed detections and localization offsets—effects that are particularly pronounced for weak splashes and distant splash events. To improve the detectability and localization stability of extremely small splashes [33], YOLO-PEGA explicitly introduces a P2/4 branch in the detection head, expanding the output scales from the conventional P3–P5 to a four-scale prediction structure with P2–P5 outputs by adding a dedicated P2 detection head (Figure 7). The core idea is as follows: in the top-down feature-fusion pathway, high-level semantic features are further upsampled and concatenated with the corresponding shallow high-resolution features from the backbone and then integrated via lightweight convolutional/residual blocks to produce a fine-grained feature map for P2-level prediction. Because the P2 feature map has a higher spatial resolution, it retains more splash-relevant edge cues and localized disturbance textures, providing richer detail support for both classification and regression and substantially alleviating the “information disappearance” of small targets in deeper feature representations.

Furthermore, introducing the P2 branch not only strengthens the representation of extremely small splashes but also improves overall consistency across splash scales. For feeding processes with large scale variations, four-scale prediction enables the network to adaptively respond at the most appropriate feature level at different stages, reducing the risk of scale mismatch that arises when relying solely on a single scale or overly deep feature levels. It should be noted that adding the P2 branch inevitably introduces additional computational and parameter overhead. However, in YOLO-PEGA, we build upon YOLO11s and incorporate subsequent structural optimizations (e.g., replacing stride-2 downsampling convolutions with ADown) to mitigate this extra burden, thereby achieving improved small-target recall and localization accuracy while preserving real-time capability.

In summary, by extending prediction scales to P2–P5, the P2 detection head allows the model to capture key splash details and boundary cues at higher resolution, enhancing the recall and localization stability of weak and small splashes. This provides a more reliable target-observation basis for subsequent feeding-intensity assessment driven by splash-detection results.

### 2.4. Feeding-Intensity Indicators

Let $f_{t}$ denote the frame-level splash-event frequency, $A_{t}\;$ the mean pixel area of the detected splash instances, and $E_{t}$ the texture intensity measure derived from grayscale/gradient energy. After detrending and min–max normalization, these variables are transformed into ${\tilde{f}}_{t}$, ${\tilde{A}}_{t}$, and ${\tilde{E}}_{t}$, respectively. The final score is defined as: $S_{t}=\alpha \;{\tilde{f}}_{t}+\beta \;{\tilde{A}}_{t}+\gamma \;{\tilde{E}}_{t}$ where α, β, and γ are obtained via grid search or learned by optimizing an objective such as maximizing correlation with expert-labeled feeding grades. Based on the time-series data in this study, fish feeding exhibits a characteristic stage-wise evolution [34].

The core idea of constructing a quantitative feeding-intensity measure from splash-monitoring results is to translate field experience-based judgments into a computable and reproducible visual indicator system. In practice, technicians and on-site experts assess the feeding status of fish schools by the intensity of water-surface disturbances during feeding, which is typically manifested as the occurrence frequency of splash events, the magnitude of individual splashes, and the roughness and high-frequency variations in surface texture. Accordingly, based on YOLO-detected splash instances, we extract three categories of visual proxy features within a unified temporal window that are most consistent with the expert criteria of “strong–moderate–weak–none,” fuse them into a continuous intensity sequence, and then map the sequence to four discrete levels.

Specifically, the continuous video is partitioned into second-level windows (or frame windows) $\left\{t\right\}$. Within each window, we compute the splash-event frequency $f_{t}$, corresponding to expert judgments of whether feeding competition is dense and whether splashing occurs persistently. We also computed a statistic of the detected bounding box pixel area $A_{t}$ (e.g., mean area or weighted mean area) to characterize the magnitude of individual splashes, reflecting whether splashing is vigorous. In addition, we introduce a texture energy indicator $E_{t}$ (derived from grayscale gradients, contrast, or energy terms within splash regions) to represent high-frequency texture disturbance intensity, thereby compensating for cases where “weak splashes” are difficult to distinguish from ripple noise based solely on bounding box counts. The three signals $\left(f_{t},A_{t},E_{t}\right)$ are then detrended and min–max normalized to obtain $\left({\tilde{f}}_{t},{\tilde{A}}_{t},{\tilde{E}}_{t}\right)$, from which a composite intensity score $S_{t}$ is constructed. The fusion weights α, β, and γ are determined via grid search or learned by maximizing consistency with expert-labeled grades (e.g., maximizing correlation, F1 score, or Cohen’s κ), ensuring that $S_{t}$ is directionally aligned with manual expert assessments. Finally, following the four-level standard provided by field experts, the continuous score $S_{t}$ is mapped to discrete levels via threshold segmentation: $S_{t}<T_{0}$ indicates no feeding (the surface is largely calm; occasional disturbances are mostly attributable to non-feeding factors); $T_{0}\leq S_{t}<T_{1}$ indicates weak feeding (sporadic splashes with low frequency and small magnitude); $T_{1}\leq S_{t}<T_{2}$ indicates moderate feeding (more continuous splashes with clearly increased frequency and magnitude but not at peak); and $S_{t}\geq T_{2}$ indicates strong feeding (dense splashing with large coverage and pronounced texture disturbance). To avoid frequent switching near thresholds, a dwell-time constraint can be imposed in practical deployment (e.g., changing the level only after it persists for Δt>τ), making the four-level decision more consistent with the continuous nature of on-site behavioral processes. Through a four-step workflow that progresses from expert knowledge to measurable feature extraction, continuous scoring, and level mapping, splash-monitoring results are standardized into an interpretable and transferable feeding-intensity criterion, providing a unified basis for automated feeding strategies and risk warning.

### 2.5. Feeding-Intensity Modeling

**Figure 8:**
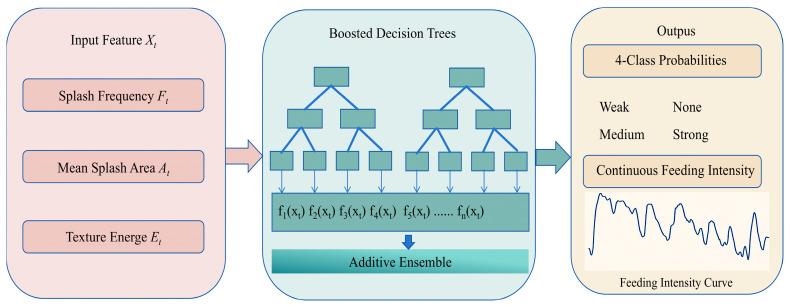
Overview of the CatBoost-based feeding-intensity modeling framework. the arrows indicate the direction of information flow.

To accurately assess fish feeding intensity, we employed a CatBoost model [35] (Figure 8) to predict continuous feeding intensity based on multiple input features. The input features include splash frequency ((F_t)), mean splash area ((A_t)), and texture energy ((E_t)), which play key roles in capturing the dynamic variations in fish activity and water-surface disturbances. Specifically, splash frequency reflects the activity level of the fish school, whereas splash area and texture energy provide quantitative descriptors of feeding-induced disturbances.

CatBoost is an efficient gradient boosting decision tree (GBDT) model [36] that adopts an additive ensemble strategy and is capable of effectively handling complex data with categorical features. In this study, CatBoost incrementally optimizes the fit to the input features through additive integration of multiple decision trees, thereby improving predictive accuracy. Each tree is trained to learn different combinations of features and contributes its prediction to the ensemble. The final model outputs the probabilities of four feeding-intensity categories (weak, moderate, strong, and no feeding).

The model outputs a probability distribution over four feeding-intensity levels and further generates a continuous feeding-intensity curve through time-series analysis, thereby characterizing feeding dynamics over time. This formulation enables not only accurate categorical classification of feeding behavior but also real-time tracking and quantification of feeding intensity, providing effective decision support for aquaculture management and environmental monitoring.

### 2.6. Model Performance Evaluation

Model performance [36] was evaluated using precision (P), recall (R), mAP@0.5, and mAP@0.5–0.95, with the corresponding formulations given in Equations (1)–(5).

(1) $$P=\frac{TP}{TP+FP}\;\;\;\;\;\;\;\;$$

(2) $$R=TP/\left(TP+FN\right)\;\;\;\;\;\;\;\;$$

(3) $$\mathrm{IoU}=\frac{\left|B\cap B_{\mathrm{pred}}\right|}{\left|B\cup B_{\mathrm{pred}}\right|}\;\;\;\;\;\;\;\;\;\;$$

(4) $${\mathrm{mAP}}_{0.5}=\frac{1}{K}\sum_{k=1}^{K}AP_{k}^{\mathrm{IoU}=0.5}\;\;\;\;\;\;\;\;\;\;\;$$

(5) $${\mathrm{mAP}}_{0.5:0.95}=\frac{1}{10K}\sum_{t=1}^{10}\sum_{k=1}^{K}AP_{k}^{\mathrm{IoU}=0.5+0.05\left(t-1\right)}\;\;\;\;\;\;\;\;$$

In Equations (1) and (2), TP, FP, and FN denote the numbers of true positives, false positives, and false negatives, respectively. Precision ((P)) measures the proportion of correctly predicted positives among all predicted positives, reflecting the reliability of model predictions. Recall ((R)) measures the proportion of true positives successfully detected among all actual positives (also referred to as sensitivity), reflecting the model’s ability to retrieve positive samples.

In object detection, Intersection over Union (IoU) is a geometric metric that quantifies the spatial overlap between a predicted bounding box and its ground truth counterpart. It is defined as the ratio of the intersection area to the union area between the predicted box (*B*_pred_) and the ground truth box (B). IoU ranges from 0 to 1; a larger IoU indicates that the prediction is closer to the ground truth in both position and scale, implying more accurate localization. Conversely, IoU values close to 0 indicate that the predicted box barely covers the target.

mAP@0.5 and mAP@0.5–0.95 denote the mean average precision computed at an IoU threshold of 0.5 and the mean across IoU thresholds from 0.5 to 0.95, respectively. These metrics provide a comprehensive evaluation of detection performance under varying localization strictness and particularly emphasize performance in high-precision localization settings.

## 3. Experimental Results

### 3.1. Experimental Setup

All experiments were conducted on an NVIDIA A100 Tensor Core GPU (NVIDIA Corporation, Santa Clara, CA, USA) to leverage its high computational throughput for accelerating deep learning model training. During certain stages, an Intel Core i9-11900K CPU (Intel Corporation, Santa Clara, CA, USA) was used for auxiliary computation to ensure stable and efficient system operation when handling CPU-bound workloads. The CPU provides 8 cores and 16 threads with a base frequency of 3.5 GHz, enabling efficient multi-threaded processing for tasks such as data preprocessing and other CPU-intensive operations.

The experimental environment was built on Ubuntu and implemented using the PyTorch (v2.1.2) framework together with the Ultralytics YOLO11 codebase for constructing and training the object detection models. CUDA acceleration was enabled to ensure efficiency and stability when processing high-resolution images and large-scale datasets. In terms of hyperparameter settings, the initial learning rate was set to 0.01 and dynamically adjusted during training. The number of training epochs was set to 300 to ensure sufficient optimization and convergence on the dataset. A batch size of 16 was used to balance training speed and memory usage, and the input image resolution was fixed at 640 × 640 to optimize computational efficiency while maintaining high detection accuracy.

### 3.2. Model Results

**Figure 9:**
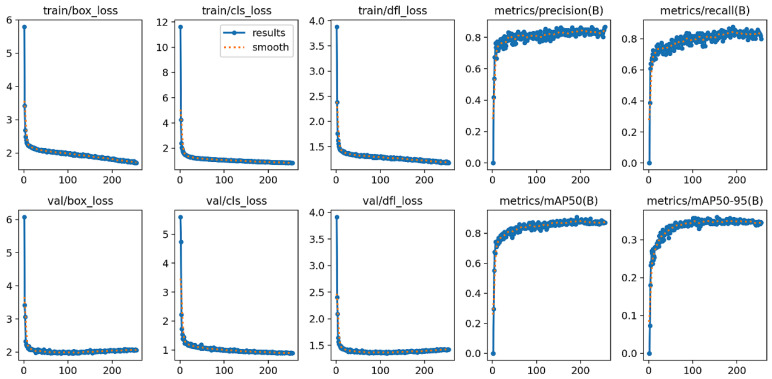
Performance of the YOLO-PEGA model on the validation set.

The results of the improved YOLO11-based model are summarized as follows (Figure 9). After training for 300 epochs, all loss terms converged smoothly, and no pronounced overfitting was observed on the validation set. On the validation set, the model achieved a precision of approximately 0.83, a recall of 0.80, an mAP@0.5 of 0.86, and an mAP@0.5:0.95 of 0.33, indicating favorable detection performance and stable convergence on this dataset.

### 3.3. Detection Results

**Figure 10:**
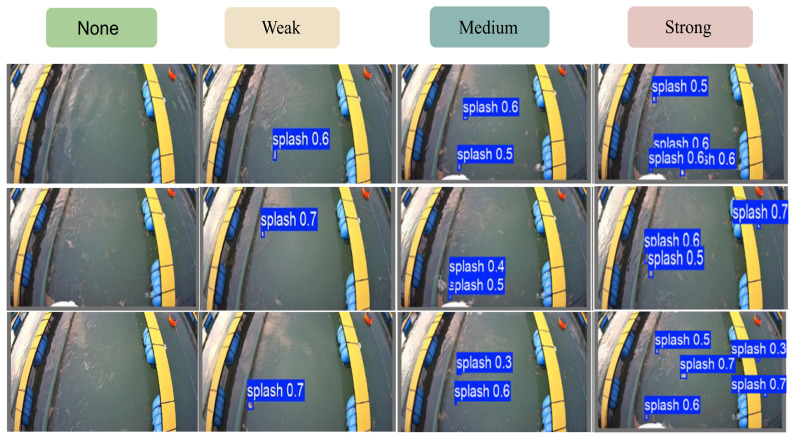
Visualization of detection results and feeding-intensity grading.

To validate the applicability of the proposed model in real-world aquaculture scenarios, we performed a visual analysis of the detection results on consecutive video frames according to the feeding-intensity grading scheme [37] (Table 2), as illustrated in Figure 10. The results indicate that the model can stably and accurately annotate “splash” targets in most frames. Predicted bounding boxes are primarily concentrated around the feed-delivery point and the adjacent disturbed water regions, and their spatial distribution is highly consistent with the true splash locations, with no obvious drift or large-scale offsets. These observations suggest that the model has effectively learned discriminative morphological and textural cues of splashes and exhibits strong perception and localization capability for splash events in the feeding area. Most detections are associated with medium-to-high confidence scores, indicating reliable recognition of splash targets.

From a temporal perspective, both the number and spatial extent of detected boxes vary synchronously with splash intensity across frames. During active feeding, the number of detections increases markedly and clusters in regions of strong surface disturbance; as feeding subsides and splashing weakens, the number of detections decreases substantially. This behavior implies that the model outputs—such as the count of “splash” instances, the average confidence score, and the spatial coverage—can reflect the dynamic progression of feeding activity and can therefore serve as effective features for quantitative feeding-intensity estimation, supporting subsequent development of automated feeding-stop and precision-feeding decision strategies.

Nevertheless, the detection results also reveal several aspects that warrant further improvement. A small proportion of predicted boxes exhibit confidence scores below 0.4, which may correspond to weak splashes or background disturbances and thus carry a risk of false positives. In addition, some splashes with small area, low color contrast, or proximity to cage boundaries were not detected, indicating occasional missed detections under complex backgrounds. Moreover, the current outputs are bounding boxes, which provide limited fine-grained delineation of splash morphology and are therefore more suitable for region-level characterization of feeding activity rather than precise shape reconstruction.

In future work, robustness and accuracy in complex aquaculture environments can be further improved by (i) augmenting the dataset with more small-object and weak-texture samples [38], (ii) optimizing the confidence threshold and non-maximum suppression (NMS) strategy, and (iii) introducing segmentation-based or cascaded refinement modules to enhance fine-grained detection performance under challenging conditions [39].

## 4. Data Analysis

### 4.1. Performance Gain Analysis

YOLO-PEGA delivers a substantial overall performance improvement for splash detection. Compared with the baseline YOLO11 model (Table 2), we observe that, while the parameter count and computational cost are markedly reduced, precision, recall, mAP@0.5, and mAP@0.5:0.95 all improve to varying degrees. This indicates that the proposed model effectively strengthens feature representation and enhances the network’s ability to discriminate and localize key splash targets despite the significant reduction in model complexity. The benefits are particularly evident under challenging conditions, including complex backgrounds with pronounced ripple interference and large variations in splash scale, where both missed detections and false alarms are mitigated.

Specifically, after introducing YOLO-PEGA, detection becomes more stable for small-scale splashes, boundary-blurred splashes, and frames containing densely overlapped multiple splashes. The predicted bounding boxes better align with target extents, and the distribution of classification confidence scores becomes more concentrated. This robustness gain on hard cases is reflected as consistent and stable improvements in aggregate metrics, and it provides a more reliable feature basis for subsequent feeding-intensity assessment based on splash frequency and spatial distribution.

### 4.2. Feeding-Intensity Analysis and Intuitive Quantification

**Figure 11:**
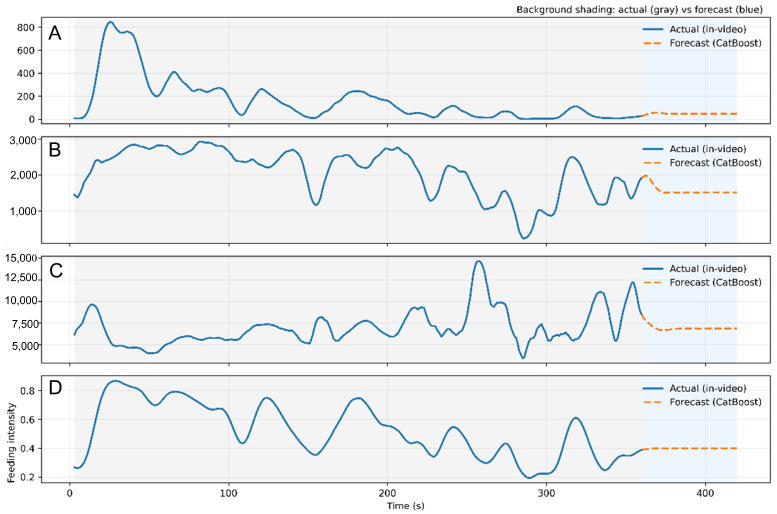
Feeding-intensity curves. (**A**) Water splash frequency displays the variation in splash frequency, peaking at the onset of feeding and gradually decreasing as feeding intensity diminishes. (**B**) Gradient energy reflects surface disturbance intensity, increasing during active feeding and declining as feeding wanes. (**C**) Texture intensity shows the change in surface texture, rising during feeding and falling as activity decreases. (**D**) Feeding intensity tepresents overall feeding intensity, peaking during active feeding and declining as the fish approach satiety.

To quantify fish feeding intensity, we analyzed the CatBoost-based predictions in conjunction with three key indicators. By comparing the observed values in the video with the CatBoost-predicted values (Figure 11), we jointly assessed feeding activity in terms of splash frequency, gradient-energy/texture cues, and the resulting composite feeding-intensity score. Splash frequency, as a primary indicator of water-surface disturbance, provides an intuitive measure of the activity intensity of fish schools during feeding. The CatBoost model demonstrates strong capability in capturing variations in splash frequency. Although the predictions exhibit a slight lag during certain abrupt transitions, the overall trend remains consistent with the observed values. Gradient energy reflects the intensity and dynamics of water-surface fluctuations, particularly the strong disturbances generated during active feeding. The experimental results show that gradient energy increases markedly during strong-feeding periods, while remaining at a low level during calm intervals. CatBoost tracks this trend effectively and provides reliable predictions; notably, during peak feeding, the predicted values closely match the observations.

Texture intensity is another important indicator that characterizes fine-grained disturbance patterns on the water surface. When fish aggregate and compete vigorously for feed, texture variations become more pronounced, leading to higher texture intensity. CatBoost captures these changes with high accuracy; although some deviations occur during phases with rapid oscillations, the predicted trajectory still reflects the underlying dynamics well.

By integrating the three indicators above, we derive a quantitative feeding-intensity value. The resulting intensity trajectory reflects the activity level and competitive strength of the fish school across different feeding stages.

During peak feeding, the estimated feeding intensity rises sharply. In particular, when splash frequency, gradient energy, and texture intensity increase concurrently, the model predictions closely match the observed values, corroborating the effectiveness and reliability of CatBoost for feeding-intensity prediction. Overall, the feeding-intensity trajectory follows a characteristic evolution pattern—rapid rise, sustained peak, and gradual decline [40]—which is highly consistent with the physiological feedback process from hunger to satiation. This provides empirical evidence for developing evaluation indicators to support intelligent feeding strategies based on feeding-behavior signals [41].

### 4.3. Feeding Strategy Recommendations

The feeding-intensity sequence $S_{t}$ derived from YOLO-based splash detection can be projected along the feeding-cycle axis to form an “intensity–time” curve, which characterizes the typical evolution of feeding behavior from an initial rapid response to a high-intensity plateau and finally to attenuation and cessation. This curve not only provides an intuitive visualization of the feeding process but also introduces a quantitative closed-loop control signal for feeding decisions. Across multiple batches of *large yellow croaker* experiments, the late-stage period in which $S_{t}$ continuously decreased and remained below a threshold $S^{*}$ for consecutive time windows was highly consistent with on-site observations that feeding had largely stopped and residual feed began to appear. Based on this pattern, an online feeding strategy can be formulated using a threshold–dwell stopping criterion:

$$S_{t}<S^{*}\;\mathrm{and}\;\Delta t>\tau \;$$

A pause in feeding (stop) is triggered, followed by an observation or cooldown window. During the τ-second period after pausing, $S_{t}$ is continuously monitored; if a pronounced rebound is observed (e.g., $S_{t}$ rises above a recovery threshold $S_{re}$, or a local peak within the window satisfies $max(S_{t})>\eta$), feeding is resumed in small, staged portions (resume). Otherwise, the system remains in the stop state until the next feeding cycle. To ensure transferability across varying illumination and hydrodynamic conditions, $S^{*}$ and τ are recommended to be learned from historical batches. For example, using the time points of “residual feed appearance” or “expert-determined feeding cessation” as labels, $S^{*}$ and τ can be determined via grid search or by maximizing correlation/consistency with the labels. In addition, hysteresis can be introduced for the recovery threshold $S_{re}$ to prevent frequent stop–start switching near the threshold.

This closed-loop strategy has several advantages: it captures the behavioral transition from hunger to satiation in real time without requiring additional sensors, reduces the risk of residual feed deposition and subsequent ammonia–nitrogen accumulation caused by overfeeding, and improves feed utilization through segmented “pause–observe–resume-on-demand” control, thereby mitigating long-term stress on the culture system induced by water-quality fluctuations.

### 4.4. Ablation Study

To quantitatively assess the contribution of each enhancement module to detection performance, we conducted a systematic ablation study using YOLO11 as the baseline (Table 3). Under strictly identical experimental settings—including dataset splits, input resolution, and training strategy (learning rate, batch size, number of epochs, confidence threshold, and NMS settings)—we varied only the enable or disable combinations of three architectural components (P2, EGMA, and ADOWN), resulting in eight model variants: YOLO11(N1),YOLO11+P2(N2),YOLO11+EGMA(N3),YOLO11+ADOWN(N4),YOLO11+P2+EGMA(N5),YOLO11+P2+ADOWN(N6),YOLO11+EGMA+ADOWN(N7),andYOLO11+P2+EGMA+ADOWN (N8).

For evaluation, we report precision (P), recall (R), mAP (IoU = 0.50), and mAP (IoU = 0.50–0.95) for each configuration, and measure inference speed in frames per second (FPS) under the same hardware conditions to assess real-time capability. The complete results are provided in Table 3, offering quantitative evidence for analyzing both the performance gains and computational costs introduced by individual modules and their combinations.

## 5. Discussion

### 5.1. Contribution of Structural Improvements to Detection Performance and Stability

Compared with the baseline YOLO11s, the proposed YOLO-PEGA introduces a structural combination of fine-grained scale compensation, edge-guided attention, and low-loss downsampling, which substantially improves detectability and training stability for splash detection under complex aquaculture backgrounds. First, the newly added P2/4 detection branch extends the prediction scales from the conventional P3–P5 to a four-scale P2–P5 design, enabling small-target regression and classification on higher-resolution feature maps. This effectively alleviates detail smoothing after repeated downsampling [42] and mitigates localization drift [43]; this is particularly critical for weak splashes, distant splashes, and splashes with fragmented boundaries. Second, by injecting EGMA into the C3k2 blocks at layers 2/4/6/8 of the backbone, the network explicitly estimates an edge-saliency map and incorporates it into channel reweighting, encouraging the model to rely more on discriminative cues such as splash boundaries, localized bright spots, and high-frequency textures, thereby reducing spurious activations induced by ripples, glare, and floating objects. Furthermore, YOLO-PEGA uniformly replaces all stride-2 downsampling convolutions in the neck/head with the proposed three-branch ADown operator, which preserves richer multi-level complementary information while reducing spatial resolution, mitigating information degradation for small targets during scale alignment and feature fusion.

Overall, the improvements in YOLO-PEGA are not achieved by simply “stacking” independent modules; rather, they constitute a targeted structural reshaping of information flow around the fundamental challenges of small-splash detection (small scale, transient appearance, fragmented boundaries, and high-frequency textures). As a result, YOLO-PEGA yields more consistent gains in precision, recall, and mAP, and improves convergence consistency across different random seeds and training durations.

### 5.2. Robustness-Oriented Design for Non-Stationary Water-Surface Conditions

The non-stationarity of water-surface conditions in aquaculture farms constitutes a major bottleneck for generalizable splash detection: variations in illumination angle and intensity can induce strong reflections and glare; wind-driven waves and aeration devices introduce surface textures that obscure splash boundaries; and changes in camera height and viewing angle further amplify differences in the target-scale distribution [44]. In response to these challenges, YOLO-PEGA is designed with a clear rationale for scene adaptation. The P2 branch provides a higher-resolution observation window that is more favorable for extremely small targets, allowing the model to retain localization-relevant details under backlighting, long-range views, or partial occlusion. EGMA performs edge-saliency-guided feature reweighting, making the network more sensitive to boundary discontinuities and splash trajectories, thereby maintaining a more stable recall in complex textured backgrounds. ADown, through three-branch complementary downsampling, reduces information collapse during scale transformations, smoothing both top-down and bottom-up feature propagation and mitigating downsampling-induced missed detections.

We note that these discussions describe the design motivation and potential mechanism of robustness. Systematic validation across different scenes, time periods, and farming pools is still required to draw definitive conclusions regarding cross-dataset generalization and on-site adaptability [45].

### 5.3. Quantifying Feeding Intensity from Splash Features and Its Behavioral Validity

Beyond detection performance, this study further transforms splash-detection outputs into a continuous behavioral signal that is actionable for feeding management, thereby closing the loop from “target recognition” to “process characterization.” Specifically, we derive three indicators within each temporal window: the frame-level splash-event frequency $f_{t}$, the mean pixel area of detected instances $A_{t}$, and the texture intensity $E_{t}$ computed from grayscale/gradient energy. After detrending and min–max normalization, these variables are converted into ${\tilde{f}}_{t}$, ${\tilde{A}}_{t}$, and ${\tilde{E}}_{t}$.

This quantification framework aligns well with the stage-wise evolution of school feeding behavior. In the early feeding stage, fish respond rapidly to exogenous feed stimuli, leading to a marked increase in splash frequency and edge-related disturbances; correspondingly, $S_{t}$ rises quickly and forms an initiation peak. The process then enters a high-intensity plateau, during which splash events become dense and their covered area increases; $S_{t}$ reaches the global maximum and remains at a high level.

As satiation feedback accumulates, splash frequency and texture energy gradually decrease; accordingly, $S_{t}$ enters a decay phase and falls below the threshold multiple times during the stopping transition. Notably, a slight rebound at the tail end is more likely to reflect school-level swimming dynamics and spatial redistribution rather than effective feeding, suggesting that intensity curves derived from splash proxies should be combined with threshold–dwell constraints and short-window decision rules to improve robustness.

Overall, YOLO-PEGA enables more reliable observation of small splashes, making splash-based intensity quantification more continuous and stable over time and providing a solid data foundation for subsequent automation and control of feeding strategies [41].

### 5.4. Interpretability Enabled by Channel Attention and Feature Selection

The structural enhancements in YOLO-PEGA also improve interpretability at the feature level to some extent. EGMA incorporates an edge-saliency map into the generation of channel attention, making the model’s responses more explicitly focused on splash boundaries and localized high-frequency textures [46]; when splashing occurs, the edge-guided branch produces stronger salient regions, thereby amplifying feature channels associated with phenomena such as abrupt bright-spot changes, fragmented textures, and boundary discontinuities [47], while relatively suppressing channels that primarily respond to large areas of stable water-surface texture. Meanwhile, the P2 branch emphasizes shallow high-resolution information, and ADown aims to preserve complementary details during scale transformations, leading to a clearer division of labor across detail preservation–semantic screening–multi-scale fusion.

Although achieving full interpretability for deep models remains challenging, these structural choices provide actionable cues for subsequent feature visualization, attention-weight analysis, and tracing the sources of specific false-positive patterns. They also offer empirical guidance for further optimizing module placement and fusion strategies.

### 5.5. Engineering Deployability and Prospects for Building a Data Ecosystem

From an engineering perspective, YOLO-PEGA is designed to achieve a deployable trade-off among accuracy, speed, and model size. Although the P2 branch introduces additional computation, the improved feature quality brought by lightweight attention on the backbone (EGMA) and low-loss downsampling during scale transitions (ADown) helps translate this overhead into tangible gains in small-target recall and localization stability. More importantly, this study establishes a relatively complete closed-loop workflow spanning data acquisition, annotation, training, online inference, and intensity estimation. As data continue to accumulate across seasons, stocking densities, and water-surface disturbance conditions, it will be possible to build a multi-scenario “splash–feeding intensity” data ecosystem and continuously improve model generalization and reliability through incremental learning or small-sample fine-tuning.

For production deployment, the threshold–dwell stopping rule based on $S_{t}$ enables quantitative control of feeding rhythm without additional sensors, with the potential to reduce residual-feed deposition and the risk of ammonia–nitrogen accumulation while improving feed utilization. In the longer term, the framework can be extended into a decision-support system for smart aquaculture that integrates multi-source information [48], providing foundational capabilities for region-scale farming management and ecological risk early warning.

## 6. Conclusions

The proposed splash–feeding mapping framework provides a practical balance among detection accuracy, real-time performance, and implementation cost. By replacing subjective experience with vision-based, detectable, and quantifiable cues, the framework supports more objective feeding-intensity assessment and has the potential to reduce residual feed and associated waste in routine aquaculture operations. The method shows robust performance on *large yellow croakers*, a regionally important cultured species. While the underlying idea may be applicable to other surface-feeding species that exhibit pronounced splashing, its generalizability across species and farming conditions warrants further systematic validation. In future work, we will extend the framework to challenging conditions. These conditions include low nighttime illumination, heavy rain, and fog. We will incorporate infrared and thermal imaging, and we will also adopt cross-sensor self-distillation, to further improve all-day reliability.

## Figures and Tables

**Table 1:** Dataset split statistics.

Dataset Split	Images	Background	Splash Instances	Image Share
Train	810	1	3250	80.0
Validation	101	1	419	10.0
Test	102	1	419	10.0
Total	1013	3	4088	100.0

**Table 2:** Feeding-intensity grading scheme.

Intensity Level	Core Quantitative Indicators	Key Distinctions	Observable Behavioral Characteristics
Strong Feeding	(1) High splash frequency; (2) large total splash area; (3) high proportion of high-confidence splashes; (4) pronounced spatial clustering of splashes.	Under strong feeding, fish compete intensely, producing persistent splashes and pronounced ripples on the water surface; accordingly, both the number and area of detected targets increase concurrently and are spatially concentrated around the feeding zone.	Multiple fish surface to compete, with frequent tail flicking and vigorous thrashing; white foam and bright spots on the surface increase; the feeding zone remains continuously disturbed with almost no “calm intervals.”
Moderate Feeding	(1) Moderate frequency; (2) moderate total area; (3) events occur in intermittent bursts, with distinct peaks but limited persistence; (4) detections remain centered on the feeding zone, with slightly increased spatial spread.	Fish remain actively feeding, but competitive intensity decreases; splash events become less frequent and occur intermittently, and the number and area of detected targets no longer stay persistently high.	Fish continue to surface but with reduced density; the water surface exhibits noticeable ripples with occasional splashes; disturbances occur in an “on–off” pattern, i.e., bursts followed by pauses.
Weak Feeding	(1) Low splash frequency; (2) Small total splash area; (3) Mostly sporadic small splashes, with a small mean area; (4) Lower confidence and reduced temporal stability of detections	Under weak feeding, the fish school shows a limited response and feeding is largely exploratory at the individual level; only minor localized surface disturbances are observed, and detected targets are few, small, and spatially dispersed.	Only a few fish surface sporadically; occasional small splashes or minor ripples are observed; the feeding zone quickly returns to a calm state, and the overall water surface remains relatively stable.
No Feeding	(1) Extremely low (near-zero) splash frequency; (2) Extremely small (near-zero) total splash area; (3) Very low proportion of high-confidence splashes; (4) Highly dispersed spatial distribution	During the no-feeding stage, the fish school exhibits little to no competitive feeding behavior, and water-surface disturbances are minimal, with almost no splashes or ripples. Accordingly, the number and area of detected targets are very small, and any splash occurrences are spatially sparse and not concentrated around the feeding zone.	The fish school remains relatively inactive, and the water surface shows virtually no discernible disturbances or activity. The feeding zone stays completely calm, with almost no detectable surface perturbations.

**Table 3:** Melting test results.

ID	P2	EGMA	ADown	Params	P	R	mAP@0.5	mAP@ [0.5:0.95]
N1	✗	✗	✗	9,413,187	0.857	0.788	0.858	0.354
N2	✓	✗	✗	9,658,340	0.854	0.794	0.869	0.369
N3	✗	✓	✗	9,628,363	0.858	0.80	0.858	0.355
N4	✗	✗	✓	8,975,299	0.857	0.8	0.865	0.363
N5	✓	✓	✗	2,715,264	0.823	0.80	0.877	0.351
N6	✓	✗	✓	2,255,612	0.844	0.81	0.883	0.359
N7	✗	✓	✓	9,190,475	0.875	0.80	0.857	0.353
N8	✓	✓	✓	2,599,772	0.868	0.80	0.883	0.359

## Data Availability

The data that support the findings of this study are available from the corresponding author upon reasonable request (subject to the permission agreement with the aquaculture enterprise/farm).
